# Supplementary material for: Dynamics of microbial competition, commensalism, and cooperation and its implications for coculture and microbiome engineering
Source: Biotechnol Bioeng. 2020 Sep 21;118(1):199–209. doi: 10.1002/bit.27562 (PMC7821011; doi:10.1002/bit.27562)
Supplement: Supplementary file 1 — Supporting information. [file BIT-118-199-s001.pdf]

Supplementary files

Dynamics of microbial competition, commensalism and cooperation and its implications for coculture and microbiome engineering

Peng Xu*

Department of Chemical, Biochemical and Environmental Engineering, University of Maryland

Baltimore County, Baltimore, MD 21250

* Corresponding author, Tel: +1(410)-455-2474; fax: +1(410)-455-1049. E-mail address:

pengxu@umbc.edu (PX)

1. Supplementary Matlab code to generate the symbolic equations in this work

```
>> syms mu_A mu_B mu_Amax mu_Bmax K_SA K_SB S S0 Y_AS Y_BS X_A X_B gamma_AB
gamma_BA P_A P_B D alpha beta k K_m Y_BA Y_PS
>> eqn1 = mu_A == mu_Amax*S/(K_SA+S)*(1+gamma_BA*X_B/(S0*Y_BS));
>> eqn2 = mu_B == mu_Bmax*S/(K_SB+S)*(1+gamma_AB*X_A/(S0*Y_AS));
>> syms X_A(t) X_B(t) S(t) P_A(t) P_B(t)
>> eqn3 = diff(X_A,t) == mu_A*X_A-D*X_A;
>> eqn4 = diff(X_B,t) == mu_B*X_B-D*X_B;
>> eqn5 = diff(S,t) == D*(S0-S) -mu_A*X_A/Y_AS -mu_B*X_B/Y_BS;
>> eqn6 = diff(P_A,t) == (alpha*mu_A+beta)*X_A-D*P_A- k*X_B*P_A/(K_m+P_A)/Y_BA;
>> eqn7 = diff(P_B,t) == k*X_B*P_A/(K_m+P_A) -D*P_B;
>> eqn8 = diff(S,t) == D*(S0-S) -mu_A*X_A/Y_AS -mu_B*X_B/Y_BS -
(alpha*mu_A+beta)*X_A/Y_PS;
```

2. Supplementary Matlab code to derive the analytical solution for Eqn1 to Eqn. 5

```
>> syms mu_A mu_B mu_Amax mu_Bmax K_SA K_SB S S0 Y_AS Y_BS X_A X_B gamma_AB
gamma_BA M1 M2 P1 P2 D alpha1 alpha2 beta1 beta2 Y_M1S Y_M2S Y_P1M1 Y_P2M2 k1 k2
K_M1 K_M2
>> eqn1 = mu_A == mu_Amax*S/(K_SA+S)*(1+gamma_BA*X_B/(S0*Y_BS));
>> eqn2 = mu_B == mu_Bmax*S/(K_SB+S)*(1+gamma_AB*X_A/(S0*Y_AS));
>> eqn3 = mu_A*X_A-D*X_A == 0;
>> eqn4 = mu_B*X_B-D*X_B ==0;
>> eqn5 = D*(S0-S) -mu_A*X_A/Y_AS -mu_B*X_B/Y_BS == 0;
```

```
>> eqns =[eqn1,eqn2, eqn3, eqn4, eqn5];
>> vars = [mu_A mu_B S X_A X_B];
>> [solmuA, solmuB, solS, solXA solXB] = solve(eqns, vars);
```

3. Supplementary Matlab code to derive the Jacobian matrix as defined by Eqn. 3-Eqn. 5

```
>> syms mu_Amax mu_Bmax K_SA K_SB S S0 Y_AS Y_BS X_A X_B gamma_AB gamma_BA D;

>> eqn1 = mu_Amax*S/(K_SA+S)*(1+gamma_BA*X_B/(S0*Y_BS))*X_A-D*X_A;

>> eqn2 = mu_Bmax*S/(K_SB+S)*(1+gamma_AB*X_A/(S0*Y_AS))*X_B-D*X_B;

>> eqn3 = D*(S0-S) - mu_Amax*S/(K_SA+S)*(1+gamma_BA*X_B/(S0*Y_BS))*X_A/Y_AS -
mu_Bmax*S/(K_SB+S)*(1+gamma_AB*X_A/(S0*Y_AS))*X_B/Y_BS;

>> JaM=jacobian([eqn1,eqn2,eqn3], [X_A,X_B,S])
```

$$Jacobian\ matrix\ =\ JaM\ =$$

$$\begin{pmatrix} \frac{S\,\mu_{Amax}}{K_{SA}+S}\left(\frac{X_B\,\gamma_{BA}}{S_0\,Y_{BS}}+1\right)-D & \frac{S\,X_A\,\gamma_{BA}\,\mu_{Amax}}{S_0\,Y_{BS}\,(K_{SA}+S)} & \frac{K_{SA}\,X_A\,\mu_{Amax}\,(S_0\,Y_{BS}+X_B\,\gamma_{BA})}{S_0\,Y_{BS}\,(K_{SA}+S)^2} \\ \frac{S\,X_B\,\gamma_{AB}\,\mu_{Bmax}}{S_0\,Y_{AS}\,(K_{SB}+S)} & \frac{S\,\mu_{Bmax}}{K_{SB}+S}\left(\frac{X_A\,\gamma_{AB}}{S_0\,Y_{AS}}+1\right)-D & \frac{K_{SB}\,X_B\,\mu_{Bmax}\,(S_0\,Y_{AS}+X_A\,\gamma_{AB})}{S_0\,Y_{AS}\,(K_{SB}+S)^2} \\ -\frac{S\,\mu_{Amax}\left(\frac{X_B\,\gamma_{BA}}{S_0\,Y_{BS}}+1\right)}{Y_{AS}\,(K_{SA}+S)}-\frac{S\,X_B\,\gamma_{AB}\,\mu_{Bmax}}{S_0\,Y_{AS}\,Y_{BS}\,(K_{SB}+S)} & -\frac{S\,\mu_{Bmax}\left(\frac{X_A\,\gamma_{AB}}{S_0\,Y_{AS}}+1\right)}{Y_{BS}\,(K_{SB}+S)}-\frac{S\,X_A\,\gamma_{BA}\,\mu_{Amax}}{S_0\,Y_{AS}\,Y_{BS}\,(K_{SA}+S)} & \frac{S\,X_A\,\mu_{Amax}\left(\frac{X_B\,\gamma_{BA}}{S_0\,Y_{BS}}+1\right)}{Y_{AS}\,(K_{SA}+S)^2}-\frac{X_A\,\mu_{Amax}\left(\frac{X_B\,\gamma_{BA}}{S_0\,Y_{BS}}+1\right)}{Y_{AS}\,(K_{SA}+S)}-\frac{X_B\,\mu_{Bmax}\left(\frac{X_A\,\gamma_{AB}}{S_0\,Y_{AS}}+1\right)}{Y_{BS}\,(K_{SB}+S)}-D+\frac{S\,X_B\,\mu_{Bmax}\left(\frac{X_A\,\gamma_{AB}}{S_0\,Y_{AS}}+1\right)}{Y_{BS}\,(K_{SB}+S)^2} \end{pmatrix}$$

Where we can simplify the Jacobian matrix by

$$J=\begin{pmatrix} A-D & C & AF \\ E & B-D & BG \\ -\frac{A}{Y_{AS}}-\frac{E}{Y_{BS}} & -\frac{B}{Y_{BS}}-\frac{C}{Y_{AS}} & -\frac{AF}{Y_{AS}}-\frac{BG}{Y_{BS}}-D \end{pmatrix}$$

$$A=\frac{S\,\mu_{Amax}}{K_{SA}+S}\left(\frac{X_B\,\gamma_{BA}}{S_0\,Y_{BS}}+1\right)$$

$$B=\frac{S\,\mu_{Bmax}}{K_{SB}+S}\left(\frac{X_A\,\gamma_{AB}}{S_0\,Y_{AS}}+1\right)$$

$$C=\frac{S\,X_A\,\gamma_{BA}\,\mu_{Amax}}{S_0\,Y_{BS}\,(K_{SA}+S)}$$

$$E=\frac{S\,X_B\,\gamma_{AB}\,\mu_{Bmax}}{S_0\,Y_{AS}\,(K_{SB}+S)}$$

$$F=\frac{K_{SA}\,X_A}{(K_{SA}+S)\,S}$$

$$G=\frac{K_{SB}\,X_B}{(K_{SB}+S)\,S}$$

4. Supplementary Matlab code to generate Fig. 6 in the manuscript

```
>> str1 = '#0072BD';
>> color1 = sscanf(str1(2:end),'%2x%2x%2x',[1 3])/255;
>> str2 = '#D95319';
>> color2 = sscanf(str2(2:end),'%2x%2x%2x',[1 3])/255;
>> str3 = '#7E2F8E';
>> color3 = sscanf(str3(2:end),'%2x%2x%2x',[1 3])/255;
>> str4 = '#77AC30';
>> color4 = sscanf(str4(2:end),'%2x%2x%2x',[1 3])/255;
>> str5 = '#4DBEEE';
>> color5 = sscanf(str5(2:end),'%2x%2x%2x',[1 3])/255;

>> subplot(2,2,1)
>> for D = 0.06:0.0025:2.0;
>> tspan = 0:0.25:500;
>> [T,Y]= ode45(@(t,y) coculture13(t,y,D),tspan, [1 1 1 1 1]);
>> yyaxis left;
>> plot(D, Y(2000,2), '.', 'Color', color1);
>> hold on;
>> plot(D, Y(2000,4), '.', 'Color', color3);
>> plot(D, Y(2000,5), '.', 'Color', color4);
>> yyaxis right;
>> plot(D, Y(2000,1), '.', 'Color', color2);
>> plot(D, Y(2000,3), '.', 'Color', color5);
>> hold on;
>> end
>> xlabel(' D (1/h)');
>> xlim([0 1.8]);
>> yyaxis left;
>> ylabel('X_A, P_A, P_B (g/L)');
>> ylim([0 10]);
>> yyaxis right;
>> ylabel('S, X_B (g/L)');
>> ylim([0 50]);
>> grid on;
>> title('(a) \gamma_{AB} = +1; \gamma_{BA} = -1');
>> hold off;

>> subplot(2,2,2);
>> for D = 0.01:0.005:2.0;
>> tspan = 0:0.25:500;
>> [T,Y]= ode45(@(t,y) coculture15(t,y,D),tspan, [1 1 1 1 1]);
>> yyaxis left;
>> plot(D, Y(2000,2), '.', 'Color', color1);
>> hold on;
>> plot(D, Y(2000,3), '.', 'Color', color5);
>> plot(D, Y(2000,4), '.', 'Color', color3);
>> plot(D, Y(2000,5), '.', 'Color', color4);
>> yyaxis right;
>> plot(D, Y(2000,1), '.', 'Color', color2);
>> hold on;
```

```
>> end
>> xlabel(' D (1/h)');
>> xlim([0 1.8]);
>> yyaxis left;
>> ylabel('X_A, X_B, P_A, P_B (g/L)');
>> ylim([0 20]);
>> yyaxis right;
>> ylabel('S (g/L)');
>> ylim([0 50]);
>> grid on;
>> title('(b) \gamma_{AB} = -1; \gamma_{BA} = +1');
>> hold off;

>> subplot(2,2,3);
>> for D = 0.01:0.005:2.0;
>> tspan = 0:0.25:500;
>> [T,Y]= ode45(@(t,y) coculture12(t,y,D),tspan, [1 1 1 1 1]);
>> yyaxis left;
>> plot(D, Y(2000,2), '.', 'Color', color1);
>> hold on;
>> plot(D, Y(2000,3), '.', 'Color', color5);
>> plot(D, Y(2000,4), '.', 'Color', color3);
>> plot(D, Y(2000,5), '.', 'Color', color4);
>> yyaxis right;
>> plot(D, Y(2000,1), '.', 'Color', color2);
>> hold on;
>> end
>> xlabel(' D (1/h)');
>> xlim([0 1.8]);
>> yyaxis left;
>> ylabel('X_A, X_B, P_A, P_B (g/L)');
>> ylim([0 25]);
>> yyaxis right;
>> ylabel('S (g/L)');
>> ylim([0 50]);
>> Legend=cell(4,1)
>> Legend{1}='X_A' ;
>>  Legend{2}='X_B';
>>  Legend{3}='P_A' ;
>>  Legend{4}='P_B';
>>  legend(Legend);
>> legend boxoff
>> grid on;
>> title('(c) \gamma_{AB} = +1; \gamma_{BA} = 0');
>> hold off;

>> subplot(2,2,4);
>> for D = 0.01:0.0025:2.0;
>> tspan = 0:0.25:500;
>> [T,Y]= ode45(@(t,y) coculture11(t,y,D),tspan, [1 1 1 1 1]);
>> yyaxis left;
>> plot(D, Y(2000,2), '.', 'Color', color1);
```

```
>> hold on;
>> plot(D, Y(2000,3), '.', 'Color', color5);
>> plot(D, Y(2000,4), '.', 'Color', color3);
>> plot(D, Y(2000,5), '.', 'Color', color4);
>> yyaxis right;
>> plot(D, Y(2000,1), '.', 'Color', color2);
>> hold on;
>> end
>> xlabel(' D (1/h)');
>> xlim([0 1.8]);
>> yyaxis left;
>> ylabel('X_A, X_B, P_A, P_B (g/L)');
>> ylim([0 16]);
>> yyaxis right;
>> ylabel('S (g/L)');
>> ylim([0 50]);
>> Legend=cell(4,1)
>>  Legend{1}='X_A' ;
>>  Legend{2}='X_B';
>>  Legend{3}='P_A' ;
>>  Legend{4}='P_B';
>>  legend(Legend);
>> legend boxoff
>> grid on;
>> title('(d) \gamma_{AB} = +1; \gamma_{BA} = +1')
```

5. Representative m. files to generate the analytical solutions and the trajectory

m.file # 1

```
function dydt = coculture1(t,y,D)
dydt=zeros(3,1);

mu_Amax=1.6; mu_Bmax=1.2; K_SA=1.0; K_SB=0.8; S0=50; Y_AS=0.5; Y_BS=0.8;
gamma_BA=-1;gamma_AB=-1;

S   = y(1);
X_A = y(2);
X_B = y(3);

% Define the two specific growth rate
mu_A = mu_Amax*S/(K_SA+S)*(1+gamma_BA*X_B/(S0*Y_BS));
mu_B = mu_Bmax*S/(K_SB+S)*(1+gamma_AB*X_A/(S0*Y_AS));

dydt(1) = D*(S0-S) -mu_A*X_A/Y_AS -mu_B*X_B/Y_BS;
dydt(2) = mu_A*X_A-D*X_A;
dydt(3) = mu_B*X_B-D*X_B;

end
```

m.file # 2

```
function dydt = coculture2(t,y,D)
dydt=zeros(3,1);


mu_Amax=1.6; mu_Bmax=1.2; K_SA=1.0; K_SB=0.8; S0=50; Y_AS=0.5; Y_BS=0.8;
gamma_BA=-1;gamma_AB=0;

S   = y(1);
X_A = y(2);
X_B = y(3);

% Define the two specific growth rate
mu_A = mu_Amax*S/(K_SA+S)*(1+gamma_BA*X_B/(S0*Y_BS));
mu_B = mu_Bmax*S/(K_SB+S)*(1+gamma_AB*X_A/(S0*Y_AS));

dydt(1) = D*(S0-S) -mu_A*X_A/Y_AS -mu_B*X_B/Y_BS;
dydt(2) = mu_A*X_A-D*X_A;
dydt(3) = mu_B*X_B-D*X_B;


end
```

m.file # 3

```
function dydt = coculture3(t,y,D)
dydt=zeros(3,1);

mu_Amax=1.6; mu_Bmax=1.2; K_SA=1.0; K_SB=0.8; S0=50; Y_AS=0.5; Y_BS=0.8;
gamma_BA=-1;gamma_AB=1;

S   = y(1);
X_A = y(2);
X_B = y(3);


% Define the two specific growth rate
mu_A = mu_Amax*S/(K_SA+S)*(1+gamma_BA*X_B/(S0*Y_BS));
mu_B = mu_Bmax*S/(K_SB+S)*(1+gamma_AB*X_A/(S0*Y_AS));

dydt(1) = D*(S0-S) -mu_A*X_A/Y_AS -mu_B*X_B/Y_BS;
dydt(2) = mu_A*X_A-D*X_A;
dydt(3) = mu_B*X_B-D*X_B;

end
```

m.file # 4

```
function dydt = coculture4(t,y,D)
dydt=zeros(3,1);


mu_Amax=1.6; mu_Bmax=1.2; K_SA=1.0; K_SB=0.8; S0=50; Y_AS=0.5; Y_BS=0.8;
gamma_BA=0;gamma_AB=-1;

S   = y(1);
X_A = y(2);
X_B = y(3);


% Define the two specific growth rate
mu_A = mu_Amax*S/(K_SA+S)*(1+gamma_BA*X_B/(S0*Y_BS));
mu_B = mu_Bmax*S/(K_SB+S)*(1+gamma_AB*X_A/(S0*Y_AS));

dydt(1) = D*(S0-S) -mu_A*X_A/Y_AS -mu_B*X_B/Y_BS;
dydt(2) = mu_A*X_A-D*X_A;
dydt(3) = mu_B*X_B-D*X_B;


end
```

m.file # 16

```
function dydt = coculture16(t,y,D)
dydt=zeros(5,1);

mu_Amax=1.6; mu_Bmax=1.2; K_SA=1.0; K_SB=0.8; S0=50; Y_AS=0.5; Y_BS=0.8;
gamma_BA=-1;gamma_AB=0;alpha=0.5;beta=0.5;Y_PS=0.4;Y_BA=0.8; k=0.8; K_m = 1.0;

S   = y(1);
X_A = y(2);
X_B = y(3);
P_A = y(4);
P_B = y(5);

% Define the two specific growth rate
mu_A = mu_Amax*S/(K_SA+S)*(1+gamma_BA*X_B/(S0*Y_BS));
mu_B = mu_Bmax*S/(K_SB+S)*(1+gamma_AB*X_A/(S0*Y_AS));

dydt(1) = D*(S0-S) -mu_A*X_A/Y_AS -mu_B*X_B/Y_BS - (alpha*mu_A+beta)*X_A/Y_PS;
dydt(2) = mu_A*X_A-D*X_A;
dydt(3) = mu_B*X_B-D*X_B;
dydt(4) = (alpha*mu_A+beta)*X_A-D*P_A- k*X_B*P_A/(K_m+P_A)/Y_BA;
dydt(5) = k*X_B*P_A/(K_m+P_A) -D*P_B;

end
```

6. Supplementary Tables: representative eigenvalues computed in this study

Representative social interactions	Dilution rate (1/h)	Steady state solutions			Three eigenvalues with respect to each steady state			Category	Stable or Unstable
		S (g/L)	X_A (g/L)	X_B (g/L)	λ_1	λ_2	λ_3		
Competition $\gamma_{AB} = -1$ $\gamma_{BA} = -1$	D = 1.0	50.000	0.000	0.000	-1.00	0.18	0.57	washout	Unstable
		1.667	24.167	0.000	-10.88	-1.00	-0.97	A survive	Stable
		4.000	0.000	36.800	-1.92	-1.00	-0.90	B survive	Stable
		25.454	3.512	14.018	-1.00	0.30	-0.33	**Coexist**	Unstable
		-2.537	10.735	24.853	-1.00	-12.25	1.11	Nonsense	Unstable
Amensalism $\gamma_{AB} = 0$ $\gamma_{BA} = -1$	D = 1.0	50.000	0.000	0.000	-1.00	0.18	0.57	washout	Unstable
		1.667	24.167	0.000	-10.88	-1.00	-0.19	A survive	Stable
		4.000	0.000	36.800	-1.92	-1.00	-0.90	B survive	Stable
		4.000	17.531	8.750	-1.00	-2.38	0.17	**Coexist**	Unstable
Parasitism $\gamma_{AB} = +1$ $\gamma_{BA} = -1$	D = 1.0	50.00	0.00	0.00	-1.00	0.18	0.57	washout	Unstable
		1.67	24.17	0.00	-10.88	-1.00	0.59	A survive	Unstable
		4.00	0.00	36.80	-1.92	-1.00	-0.90	B survive	Stable
		0.05	312.08	-459.4	-1.00	-1027.61	-0.62	Nonsense	Stable
		39.53	-3.75	14.37	-1.00	0.31	-0.32	Nonsense	Unstable
Parasitism $\gamma_{AB} = +1$ $\gamma_{BA} = -1$	D = 1.365	50.00	0.00	0.00	-1.37	-0.18	0.20	washout	Unstable
		5.81	22.10	0.00	-1.53	-1.37	0.62	A survive	Unstable
		-6.62	0.00	45.29	-1.61	-1.61	-1.37	Nonsense	Stable
		0.08	289.05	-422.5	-1.37	-0.74	-943.44	Nonsense	Stable
		35.70	4.07	4.92	-1.37	**- 0.0068 + 0.19*i***	**-0.0068 - 0.19*i***	**Coexist**	**Stable**
Amensalism $\gamma_{AB} = -1$ $\gamma_{BA} = 0$	D = 1.0	50.00	0.00	0.00	-1.00	0.18	0.57	Washout	Unstable
		1.67	-5.83	48.00	-1.00	-8.76	-0.29	Nonsense	Stable
		1.67	24.17	0.00	-10.88	-1.00	-0.97	A survive	Stable
		4.00	0.00	36.80	-1.92	-1.00	0.28	B survive	Unstable
Neutralism $\gamma_{AB} = 0$ $\gamma_{BA} = 0$	D = 1.0	50.00	0.00	0.00	-1.00	0.18	0.57	washout	Unstable
		1.67	24.17	0.00	-10.88	-1.00	-0.19	A survive	Stable
		4.00	0.00	36.80	-1.92	-1.00	0.28	B survive	Unstable
Commensalism $\gamma_{AB} = +1$ $\gamma_{BA} = 0$	D = 1.0	50.000	0.000	0.000	-1.00	0.18	0.57	Washout	Unstable
		1.667	5.833	29.333	-1.00	-9.60	-0.16	**Coexist**	**Stable**
		1.667	24.167	0.000	-10.88	-1.00	0.59	A survive	Unstable

		4.000	0.000	36.800	-1.92	-1.00	0.28	B survive	Unstable
Parasitism $\gamma_{AB} = -1$ $\gamma_{BA} = +1$	D = 1.0	50.00	0.00	0.00	-1.00	0.18	0.57	washout	Unstable
		1.67	24.17	0.00	-10.88	-1.00	-0.97	A survive	Stable
		4.00	0.00	36.80	-1.92	-1.00	1.46	B survive	Unstable
		-0.03	487.78	-740.4	-1.00	-1.56	1254.78	Nonsense	Unstable
		60.45	3.89	-14.59	-1.00	0.18	0.57	Nonsense	Unstable
Commensalism $\gamma_{AB} = 0$ $\gamma_{BA} = +1$	D = 1.0	50.00	0.00	0.00	-1.00	0.18	0.57	Washout	Unstable
		1.67	24.17	0.00	-10.88	-1.00	-0.19	A survive	Stable
		4.00	0.00	36.80	-1.92	-1.00	1.46	B survive	Unstable
		4.00	28.47	-8.75	-1.00	-2.64	0.25	Nonsense	Unstable
Cooperation $\gamma_{AB} = +1$ $\gamma_{BA} = +1$	D = 1.0	50.00	0.00	0.00	-1.00	0.18	0.57	Washout	Unstable
		1.67	24.17	0.00	-10.88	-1.00	0.59	A survive	Unstable
		4.00	0.00	36.80	-1.92	-1.00	1.46	B survive	Unstable
		76.24	-3.95	-14.67	-1.00	-0.33	0.33	Nonsense	Unstable
		0.85	15.51	14.51	-1.00	-29.92	-0.30	**Coexist**	**Stable**
Cooperation $\gamma_{AB} = +1$ $\gamma_{BA} = +1$	D = 1.365	50.00	0.00	0.00	-1.37	-0.18	0.20	Washout	Unstable
		5.81	22.10	0.00	-1.53	-1.37	0.62	A survive	Unstable
		-6.62	0.00	45.29	-1.61	-1.37	2.65	Nonsense	Unstable
		48.66	3.91	-5.17	-1.37	**- 0.0007 + 0.19*i***	**-0.0007 - 0.19*i***	Nonsense	Stable
		1.81	15.99	12.96	-1.37	-0.39	-11.92	**Coexist**	**Stable**
Cooperation $\gamma_{AB} = +1$ $\gamma_{BA} = +1$	D = 1.60	50.00	0.00	0.00	-1.60	-0.42	-0.03	**Washout**	Stable
		-3.20	0.00	42.56	-8.87	-1.60	3.20	Nonsense	Unstable
		29.87	9.23	1.34	-1.60	0.12	-0.16	**Coexist**	Unstable
		3.46	16.04	11.56	-1.60	-4.17	-0.41	**Coexist**	**Stable**
Cooperation $\gamma_{AB} = +1$ $\gamma_{BA} = +1$	D = 1.70	50.00	0.00	0.00	-1.700	-0.519	-0.131	**Washout**	Stable
		-17.00	33.50	0.00	-1.700	-0.419	1.247	Nonsense	Unstable
		-2.72	0.00	42.18	-13.729	-1.700	3.498	Nonsense	Unstable
		20.75	11.78	4.55	-1.700	0.218	-0.324	**Coexist**	Unstable
		5.29	15.77	10.53	-1.700	-0.381	-1.785	**Coexist**	**Stable**
